# Towards the Future of Cancer Care: A Concept Paper for the Proposal of a Medical and Public Health Cancer Genomics Platform

**DOI:** 10.1002/hsr2.73055

**Published:** 2026-08-18

**Authors:** M.R. Cozzolino, M.G. Cacciuttolo, A. Ammendolia, R. Pastorino, S. Boccia, M.L. Specchia

**Affiliations:** ^1^ Section of Hygiene, University Department of Life Sciences and Public Health Università Cattolica del Sacro Cuore Rome Italy; ^2^ Fondazione Policlinico Universitario A. Gemelli IRCCS Rome Italy

**Keywords:** cancer care, cancer genomics, public health, web platform

## Abstract

**Background and Aims:**

This study aims to develop the concept of a medical and public health cancer genomics platform for aggregating and sharing documentation of ongoing initiatives and data, available for EU researchers, healthcare professionals, policy makers, citizens and patients.

**Methods:**

The context, feasibility, and potential end users of the platform were analysed; project deliverables were identified and clustered; and aspects related to the definition of the overall platform architecture and the integration of its modules were investigated.

**Results:**

To define the overall system architecture should be implemented: selection of a content management system, hosting configuration and development domain setup, content management system installation, development of HTML web pages, data entry and visualisation; graphic layout design, and testing and debugging. To support modules integration, the following activities should be addressed: creation of a server analytics account; integration with social networks; and activation of a newsletter system.

**Conclusion:**

Cloud‐based data platform enable the securely analysis and sharing of large datasets. Establishing an infrastructure to facilitate researchers' access to and storage of large volumes of biological data is of paramount importance, both to advance research and to support health policy‐making processes. An online platform could help align clinical and population‐based interventions and support the integration of the Genome of Europe biobanking initiative into public health genomics for cancer.

## 1 Introduction

The amount of data collected in biomedical research has increased significantly over time, mainly due to the development of powerful new research technologies [1]. Although the large and continuously growing body of evidence produced in medicine and healthcare is well documented, science remains effectively data‐limited, as available data must be carefully managed and harmonised to be truly useful [2].

Connecting healthcare data and securely integrating it with additional open and social data sources can support not only research activities but also policy and decision‐making processes, ensuring secure access to relevant information to effectively promote population health and wellbeing [3]. Clinical research informatics tools have expanded the possibilities for scientific discovery by providing researchers with new perspectives and reproducible approaches for visualising and analysing heterogeneous data [4, 5, 6]. Moreover, digital communication has become a major source of scientific and medical information for healthcare professionals [7].

Currently, researchers use a wide range of tools and platforms to store and analyse biological data, including whole‐genome sequencing data, advanced imaging studies, comprehensive proteomic analyses, and detailed clinical annotations [1]. A “data commons” is described in the literature as a cloud‐based data platform that enables a community to securely manage, analyse, and share large datasets through cloud computing, thereby accelerating research [8]. However, advances in science and healthcare delivery, as well as improvements in health outcomes, are strongly dependent on effective data sharing [9].

This is particularly relevant in the field of oncology, where cancer data are often fragmented and compartmentalised. As a result, many stakeholders are working to overcome the challenges this fragmentation poses to research progress. To improve and accelerate advancements, researchers need access to data from multiple institutions. Establishing an infrastructure that facilitates access to, integration, storage, and analysis of large volumes of biological data and related information is therefore of paramount importance [1], both to enable research progress and to support health policymaking processes [3]. According to Ghezzi et al. [10], desirable qualities of software products and processes include correctness, reliability, performance, usability, verifiability, maintainability, repairability, evolvability, reusability, portability, understandability, interoperability, productivity, visibility, and timeliness.

This concept paper arises from activities conducted within the “Building the EU Cancer and Public Health Genomics Platform” (CAN.HEAL) project, which responds to the lack of international multicentric collaboration needed to advance cancer prevention, diagnosis, and treatment on a large scale. The clinical work of the project focuses on the application of next‐generation sequencing technologies and on identifying implementation pathways to extend the use of genetic profiling of patients and tumour cells.

One of the project's tasks was the establishment of a Medical and Public Health Cancer Genomics Platform for health research and healthcare purposes, aimed at supporting the responsible and effective translation of genome‐based knowledge and technologies into public policy and health services for the benefit of population health [11].

Beyond the specific objectives of CAN.HEAL, this concept proposes the structure and key attributes of an open‐access Medical and Public Health Cancer Genomics Platform designed to support European research projects in this field. The platform would enable the aggregation and sharing of cancer care data and make research‐derived evidence and knowledge available to researchers, healthcare professionals, policymakers, as well as citizens and patients.

This manuscript presents a conceptual architecture rather than defining a fixed operational dataset. The platform is intended to accommodate genomic summary information (e.g., variant frequencies), aggregate transcriptomic profiles, clinical phenotypes, epidemiological indicators, and biobank metadata. The specific scope, level of granularity, and admissibility of datasets should be determined at the project level and in accordance with applicable regulatory, ethical, and governance frameworks. The architecture is designed to be modular and replicable, enabling adaptation across different European contexts and allowing potential future evolution toward federated or distributed models, if required.

## 2 Methods

**Figure 1:**
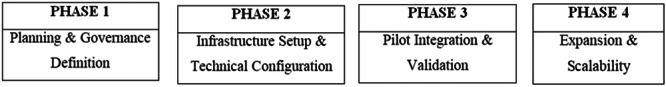
Platform development timeline.

Inspired by comparable projects that aimed to create the architecture of a European platform for health policy decision‐making [3] and to combine deidentified patient clinical data and pathogen genomic variants [4], in order to develop the platform, the following phases are proposed (Figure 1).

The methodological steps for the development of the web platform are reported and described below.

### 2.1 Preliminary Activities

A series of internal working group meetings and brainstorming sessions should be organised with the aim of creating the basis for the platform development activities, based on the research project aims.

An example of a brainstorming tools could be a collaborative whiteboard (ideal for workshops and remote teamwork) that could allow structured brainstorming with voting and timers, an infinite canvas, digital sticky notes, ready‐made templates and real‐time collaboration. The working group should be composed of: a platform project manager to coordinate development, the timeline, and data quality; a scientific lead to design the data structure and modules; end‐user representatives to validate functionality and usability; an IT technical lead to build the platform architecture; software engineers to implement modules and APIs; and security & privacy experts to guarantee data protection and GDPR compliance. In this phase, context and feasibility analyses should be performed.

*Context analysis:* The context in which the project is to be developed should be carefully assessed, including aspects related to the reference sector, relevant stakeholders, and the available scientific evidence. The reference sector is generally represented by public health genomics, which has been defined as the “responsible and effective translation of genome‐based discovery into population health” [12]. However, it should be more precisely defined on a case‐by‐case basis, depending on the specific objectives of each project. In public health genomics, the target audience consists of multiple stakeholder groups involved in cancer care, including patients—who are at the centre of the care pathway—as well as researchers, clinicians, and decision‐makers [13]. Available scientific evidence indicates that advances in science and healthcare, as well as improvements in health outcomes, are closely linked to effective data sharing. Overcoming the compartmentalisation of cancer data to promote research progress therefore represents a major challenge for many stakeholders [1;9].

*Feasibility analysis:* The implementation timeline and resources required for the development of the web platform [14, 15] should be analysed in alignment with the overall project schedule. The necessary human resources should be identified among the professionals involved in the project working groups, including senior public health experts, junior researchers, computer science engineers, and other specialists relevant to the project's specific focus. The data and materials produced by all project work packages (WPs) should be identified as the core content of the platform. Particular attention should be given to privacy management. With regard to data sharing, and in accordance with Regulation (EU) 2016/679 of the European Parliament and of the Council on the protection of natural persons with regard to the processing of personal data, as well as subsequent updates [16], patient data from different sources should be linked in a manner that preserves privacy and prevents the disclosure of identifiable information. Indeed, the management, disclosure, and protection of personal information represent one of the most important—yet challenging—social processes in everyday life [17].

**Figure 2:**
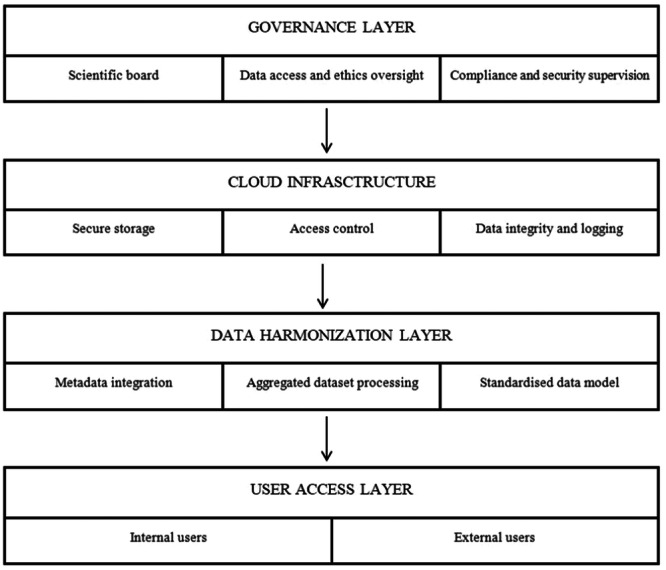
Organizational diagram.

The platform organizational development can be summarized as shown in Figure 2.

### 2.2 Analysis of End Users of the Platform

Participants involved in the project and the relevant stakeholders should be carefully analysed. The platform's end users can be divided into internal and external users. Internal users include the experts who are part of the project consortium and who contribute to producing the data and documents shared through the platform. External users include patients, clinicians, researchers, health policymakers and decision‐makers, as well as other individuals who are not directly involved in the project but are considered stakeholders. Internal users should be granted access to a broader range of information than external users. To maintain organisational efficiency and minimise the exposure of sensitive information, systems, or data, it is essential to define access rights based on users' specific roles and needs [18]. To this end, an internal governance procedure should be established and shared with the project's principal investigator and the individuals responsible for communication and dissemination activities. This procedure should clearly define the platform's governance framework, including: management of data input and metadata; data quality control procedures; dataset submission processes (e.g., merging datasets and preventing duplicate samples); specification of which data will be accessible to internal versus external users and under what conditions; identification of individuals responsible for communication and dissemination; and standardisation and monitoring of the document upload process to ensure that all data are properly stored within the platform.

### 2.3 Analysis and Clustering of the Project Results

Through regular checks and the analysis of the documents produced by the different project WPs, the results/deliverables obtained should be catalogued into categories.

The different result/deliverable categories thus identified could represent the main labels of the cloud interface and guide the architecture development of the platform. These could be summarised, for example, as follows:

- Results from events
- Papers and protocols
- Newsletters
- Mapping results and reports
- Datasets
- Recommendations and guidelines
- Use cases
- Courses and training activities

### 2.4 Definition of the Overall System Architecture

A centralized cloud and edge computing platform shall be developed with a data centre providing all the technological services, allowing for high flexibility and availability. Users could access the cloud services and data remotely through the web [19]. The development of data storage and network interfaces shall be planned, as well as the analysis of the platform end‐users.

To define the overall system architecture, the following aspects should be considered [20, 21]:

#### 2.4.1 Content Management System (CMS) Choice

The CMS should be open source and cloud‐based. Access to the platform should be achieved through Hypertext Transfer Protocol Secure (HTTPS) and be cryptographically secure [22, 23]. The CMS needs to protect the users' credentials as well as the application's backend infrastructure [24].

#### 2.4.2 Hosting Configuration and Setup of the Development Domain

Hosting is the service that allows all the files that make up the site to be physically hosted on a web server, thus making the site accessible via the Internet. The development of the project platform will require the use of a private “development domain”, where all the work, verification, and test phases will be carried out.

#### 2.4.3 Content Management System (CMS) Installation

The CMS is a software tool whose task is to facilitate the management of website contents, freeing the user from the need for specific technical knowledge of web programming. Technically, a CMS is a server‐side application that relies on a database for content archiving.

The application is divided into two parts:

– an administration section (back end), to organise and supervise content production;
– an application section (front end), through which end users can access the contents and site applications.
  The CMS will be:
– in English;
– intuitive and intended to be used also by non‐medical personnel;
– compliant with the main web security requirements, to avoid threats in communications, devices/services, users, mobility and integration of resources [25];
– usable from mobile devices (i.e., tablets and smartphones).

The chosen system architecture is based on the LAMP stack (Linux ‐ Apache ‐ MySQL ‐ PHP) and Open Source technology. The Linux server will include a web Server APACHE; PHP as the software processing “engine”; and MySQL as the Database Management System. After the CMS installation, the information structure will be created to contain the shared content. Subsequently, the chosen CMS must be appropriately customized based on the purposes of the project, through the implementation of the necessary forms and functions. This will have an impact on the entire architecture of the system: from the database to the application modules, which will have to be appropriately designed according to the guidelines of the chosen CMS [20, 21].

#### 2.4.4 Development of HTML Web Pages With Responsive Technology

It should be considered that the platform can be accessed and consulted through many devices differing in screen size and resolution (smartphones, tablets, notebooks, PCs, and even televisions) and, in the case of smartphones and tablets can be used in both a vertical (Portrait) and a horizontal (Landscape) orientation. Therefore, a Responsive Design technology consisting of the ability to adapt the display to different devices will have to be used. The implementation of an app is not planned, but the CMS will also be usable on mobile devices as the HTML interfaces will be developed with responsive technology [22, 23].

#### 2.4.5 Data Entry

Special attention should be given to the quality of the textual contents, the organisation of the materials and the method of presenting them. Given the importance of the content insertion phase, particular attention should be paid to:

– text analysis and use of appropriate terminology;
– review of the contents proposed in the brainstorming phase.

#### 2.4.6 Data Visualisation and Graphic Layout

The website layout encompasses all elements related to the organisation and presentation of content, including the structure of individual pages, the logo, and the overall graphic design. Graphic design, in particular, is one of the aspects that most directly influences the user experience during navigation. Therefore, special attention should be paid to developing an interface that is both effective and intuitive, ensuring ease of use and accessibility [20].

Authorised users should be granted access to a cloud‐based environment that allows them to upload, manage, and consult the data and materials produced by the various project research groups. All other users should be granted read‐only access, enabling them to consult the data and materials made available on the platform without the ability to modify or upload content.

#### 2.4.7 Test and Debug

The most natural and traditional way to test a product is to try it in a certain number of representative situations, ensuring that it behaves as expected. In general, it is impossible to test software in all its possible operating conditions and it is therefore necessary to find some test cases that provide sufficient evidence that the product will have acceptable behaviour even in situations in which it has not been tested. The goal of testing is to identify errors (bugs) in programs. The term *debugging* indicates the activity of identifying and correcting errors and begins every time a malfunction is discovered [10].

Testing will include staged validation phases:

– internal alpha testing by consortium members;
– controlled beta testing involving selected external institutions;
– cybersecurity validation, including access control verification and penetration testing where appropriate.

A structured ticket‐based reporting system will allow users to report malfunctions or usability issues. All technical updates and corrections will be documented and managed through version‐controlled releases to ensure transparency and traceability.

### 2.5 Modules Integration

The following items should be addressed [20, 21]:

#### 2.5.1 Creation of a Server Analytics Account

A server analytics account should be created to analyse detailed statistics on the platform's external users by clustering, concept searching, and categorization. The use of the server will make it possible to identify the most viewed platform pages/contents, monitor user volume, number of accesses, and time spent using the platform, and categorize users, both individually and in groups, based on their characteristics (e.g., institutions/bodies they belong to, professional qualification, geographical location).

#### 2.5.2 Website and Social Network Integration

The platform could be linked to:

– the project website (the platform will allow users to store documents and materials and to explore topics cited on the website in greater depth);
– social media pages;
– e‐mail accounts.

Social media and website integration refers exclusively to communication and dissemination activities (e.g., sharing updates, events, newsletters, and public information materials). No social media data will be integrated into clinical, genomic, or research datasets hosted within the platform.

#### 2.5.3 Activation of Additional System for Sending Newsletters

An email service should be activated with a dedicated mailbox to facilitate communications related to the use of the platform. In addition, the platform could be configured to support the distribution of newsletters to specific user groups. It should also allow the tracking of all emails sent, the monitoring of delivery and engagement statistics for each group, and the comparison of audience responses to different communications. For each newsletter, the system could record metrics such as which users opened the email, viewed embedded images, and clicked on links included in the message.

### 2.6 Governance and Harmonised Data Sharing Framework

The platform should operationalise national regulatory diversity through a federated infrastructure, harmonised governance, and international technical standards, enabling secure and GDPR‐compliant genomic data sharing across Europe:

– Regulatory and Policy Alignment
  Genomic data qualify as personal data and as a special category of data under the General Data Protection Regulation (GDPR), requiring enhanced safeguards, a lawful basis for processing (Art. 9), and appropriate research‐specific protections (Art. 89) [16]. While respecting national specificities in governance and consent, it will apply a structured interoperability model to manage legal fragmentation and ensure compliance.
– Federated Organisational Model
  A federated architecture will keep data within national infrastructures, enable secure cross‐border analysis through a compute‐to‐data approach, and avoid unnecessary transfers. This strengthens trust, respects data sovereignty, and reduces barriers.
– Policy Harmonisation Mechanisms
  Legal interoperability will be supported through standardised Data Use Agreements, common access procedures, harmonised consent templates, risk‐based data classification, and a GDPR Code of Conduct for genomic data sharing.
– Data Harmonisation and Interoperability

The platform will support heterogeneous data formats (e.g., structured clinical data, genomic summary data, epidemiological indicators, and imaging‐related metadata) through a structured submission interface. Rather than storing raw patient‐level genomic files, the platform will prioritise the integration of harmonised metadata and aggregated datasets that are not directly identifiable and are compliant with EU data protection regulations.

A secure cloud‐based European infrastructure will ensure controlled access, scalability, and data integrity.

As already said, this manuscript proposes a conceptual architecture of a platform and does not describe specific operational steps. For this reason, concrete mechanisms (e.g., common data model, standard vocabularies, format‐mapping process) for harmonising heterogeneous data types and file formats into a cohesive dataset have not been analysed. This specific aspect will have to be addressed in a future technical and operational phase.

### 2.7 Set up of Training Courses for End Users

In order to allow end‐users to familiarise themselves with the platform and easily share/download documents, explanatory materials should be produced (e.g., PDF files, PowerPoint slides, videos) and shared with relevant internal and external stakeholders. Feedback from end users could be collected through online surveys. The timing of this feedback request during the platform development will have to be determined at a later project stage.

### 2.8 Platform Sustainability

The long‐term sustainability should rely on institutional embedding within European research ecosystems and alignment with emerging EU initiatives such as the European Health Data Space.

Operational sustainability will require dedicated IT personnel, governance oversight mechanisms, cybersecurity monitoring, and periodic infrastructure updates.

The modular and replicable architecture of the platform facilitates adaptation and reuse across projects, enhancing long‐term viability. Future funding mechanisms may include European research programmes, institutional partnerships, and integration with existing data infrastructures.

## 3 Conclusion

This paper proposes the concept of a Medical and Public Health Cancer Genomics Platform to support European research projects by aggregating and sharing cancer care data and making research‐derived evidence and knowledge accessible to all relevant stakeholders. The concept outlines the overall system architecture, the main elements of the cloud interface, and the planned integration of modules. The cloud‐based platform is intended to securely manage, integrate, analyse, and share large datasets.

The proposed platform aligns with the EU Health Policy Platform (https://webgate.ec.europa.eu/hpf/), a collaborative online tool that allows European Commission services and health‐related interest groups to discuss public health concerns and provides an efficient means for stakeholders to share knowledge and best practices. Members can post news, create opinion polls, share upcoming events or webinars, store documents, and more across one or multiple networks on the platform [26].

Securely connecting healthcare data and integrating it with additional open and social data can help overcome the challenge of safely accessing all relevant information for policy and decision‐making processes, ultimately supporting population health and wellbeing. Establishing an infrastructure that allows researchers to access, store, and analyse large volumes of biological data is therefore critical to advancing research and informing health policy.

The proposed Medical and Public Health Cancer Genomics Platform can also provide guidance for reducing barriers to the adoption of personalised medicine at the EU level, representing an opportunity to improve individualised healthcare for all citizens and offering substantial promise for disease treatment and prevention [27]. Beneficiaries include cancer patients and the general public, healthcare professionals, researchers, and policymakers. Patients and citizens will benefit from more effective clinical support, healthcare professionals and researchers will gain a broader understanding of oncological diseases and associated risks, and policymakers will receive recommendations for integrated systems aimed at reducing the cancer burden and improving the population's quality of life.

As the initiative develops, the target population is expected to expand to include the broader health community and policymakers beyond the healthcare sector. A platform such as the one proposed in this concept paper can help align clinical and population‐based interventions and integrate the Genome of Europe biobanking initiative into public health genomics for cancer.

## Author Contributions

**M.R. Cozzolino:** writing – original draft, investigation, methodology, conceptualization, project administration. **M.G. Cacciuttolo:** investigation, methodology. **A. Ammendolia:** conceptualization. **R Pastorino:** writing – review and editing. **S. Boccia:** writing – review and editing. **M.L. Specchia:** supervision, validation, writing – review and editing, project administration.

## Conflicts of Interest

The authors declare no conflicts of interest.

## Declaration

All authors have read and approved the final version of the manuscript. Maria Rosaria Cozzolino had full access to all of the data in this study and takes complete responsibility for the integrity of the data and the accuracy of the data analysis. She also affirms that this manuscript is an honest, accurate, and transparent account of the study being reported; that no important aspects of the study have been omitted; and that any discrepancies from the study as planned have been explained.

## Data Availability

The authors confirm that the data supporting the findings of this study are available within the article and its supplementary materials.
